# Point-of-care echocardiography screening for hypertrophic cardiomyopathy using automated deep-learning analysis

**DOI:** 10.1093/ehjdh/ztag140

**Published:** 2026-09-09

**Authors:** Nour Karra, Yarin Klempfner, Viana Copeland, Michael Fiman, Harel Doitch, Roei Merin, Robert Klempfner, Ehud Schwammenthal, Michael Arad, Elad Maor

**Affiliations:** Leviev Heart and Vascular Center, Chaim Sheba Medical Center, Derech Sheba 2, Tel Hashomer, 52621 Ramat Gan, Israel; The Gray Faculty of Medical & Health Sciences, Tel Aviv University, Ramat Aviv, 69978 Tel Aviv, Israel; AISAP.ai, Derech Sheba 2, 5266202 Ramat Gan, Israel; Leviev Heart and Vascular Center, Chaim Sheba Medical Center, Derech Sheba 2, Tel Hashomer, 52621 Ramat Gan, Israel; AISAP.ai, Derech Sheba 2, 5266202 Ramat Gan, Israel; AISAP.ai, Derech Sheba 2, 5266202 Ramat Gan, Israel; The Gray Faculty of Medical & Health Sciences, Tel Aviv University, Ramat Aviv, 69978 Tel Aviv, Israel; Tel Aviv Sourasky Medical Center, Affiliated to the Gray Faculty of Medical & Health Sciences, Tel Aviv University, Israel; Leviev Heart and Vascular Center, Chaim Sheba Medical Center, Derech Sheba 2, Tel Hashomer, 52621 Ramat Gan, Israel; AISAP.ai, Derech Sheba 2, 5266202 Ramat Gan, Israel; The Gray Faculty of Medical & Health Sciences, Tel Aviv University, Ramat Aviv, 69978 Tel Aviv, Israel; AISAP.ai, Derech Sheba 2, 5266202 Ramat Gan, Israel; Leviev Heart and Vascular Center, Chaim Sheba Medical Center, Derech Sheba 2, Tel Hashomer, 52621 Ramat Gan, Israel; The Gray Faculty of Medical & Health Sciences, Tel Aviv University, Ramat Aviv, 69978 Tel Aviv, Israel; Leviev Heart and Vascular Center, Chaim Sheba Medical Center, Derech Sheba 2, Tel Hashomer, 52621 Ramat Gan, Israel; The Gray Faculty of Medical & Health Sciences, Tel Aviv University, Ramat Aviv, 69978 Tel Aviv, Israel; AISAP.ai, Derech Sheba 2, 5266202 Ramat Gan, Israel

**Keywords:** Hypertrophic cardiomyopathy, Echocardiography, Point of care, Screening, Deep learning

## Abstract

**Aims:**

Hypertrophic cardiomyopathy (HCM) remains underdiagnosed due to limited access to expert imaging. We developed and validated a deep-learning (DL)-based echocardiographic model adaptable to point-of-care ultrasound (POCUS) for scalable HCM screening.

**Methods and results:**

We retrospectively analysed 134 956 expert transthoracic echocardiograms (TTE) from 73 598 patients at Sheba Medical Center (2007–2022). A TTE-trained DL model integrating structural features and temporal motion patterns from parasternal long-axis and apical four-chamber views estimated HCM probability. Performance was evaluated in an independent test cohort and clinical subgroups. External validation used bedside POCUS studies from non-cardiologists with handheld devices. The test cohort included 12 096 patients with 119 confirmed HCM cases (prevalence 0.98%; median age 75 years, 57% male). HCM-positive patients showed increased expert TTE-measured septal (1.67 [1.5, 2.0] vs. 1.01 [0.9, 1.19] cm) and posterior wall thickness (1.1 [1.0, 1.3] vs. 0.9 [0.8, 1.0] cm) (*P* < 0.001). The model achieved excellent discrimination with an area under the curve of 0.982 (95% CI 0.966–0.993), sensitivity 88.2%, and specificity 97.3%, robust across subgroups. The POCUS cohort (*n* = 1047, median age 73 years, 55% male) represented multimorbid inpatients with 65 (6.2%) classified as screen-positive by the algorithm. These showed higher expert TTE-measured septal thickness (1.26 [1.07, 1.46] vs. 1.06 [0.9, 1.2] cm; 22% vs. 4% with IVS ≥1.5 cm; *P* ≤ 0.01). Among 49 (75%) POCUS-flagged positive patients with formal TTE and clinical data, 8 (16%) were confirmed by expert adjudication to have HCM. Specificity is limited by occasional confounding amyloidosis detection (4% of POCUS-flagged patients).

**Conclusion:**

This DL-based model identifies HCM and demonstrates feasibility for POCUS screening, supporting earlier detection and broader diagnostic access.

## Graphical Abstract

**Graphical Abstract:**
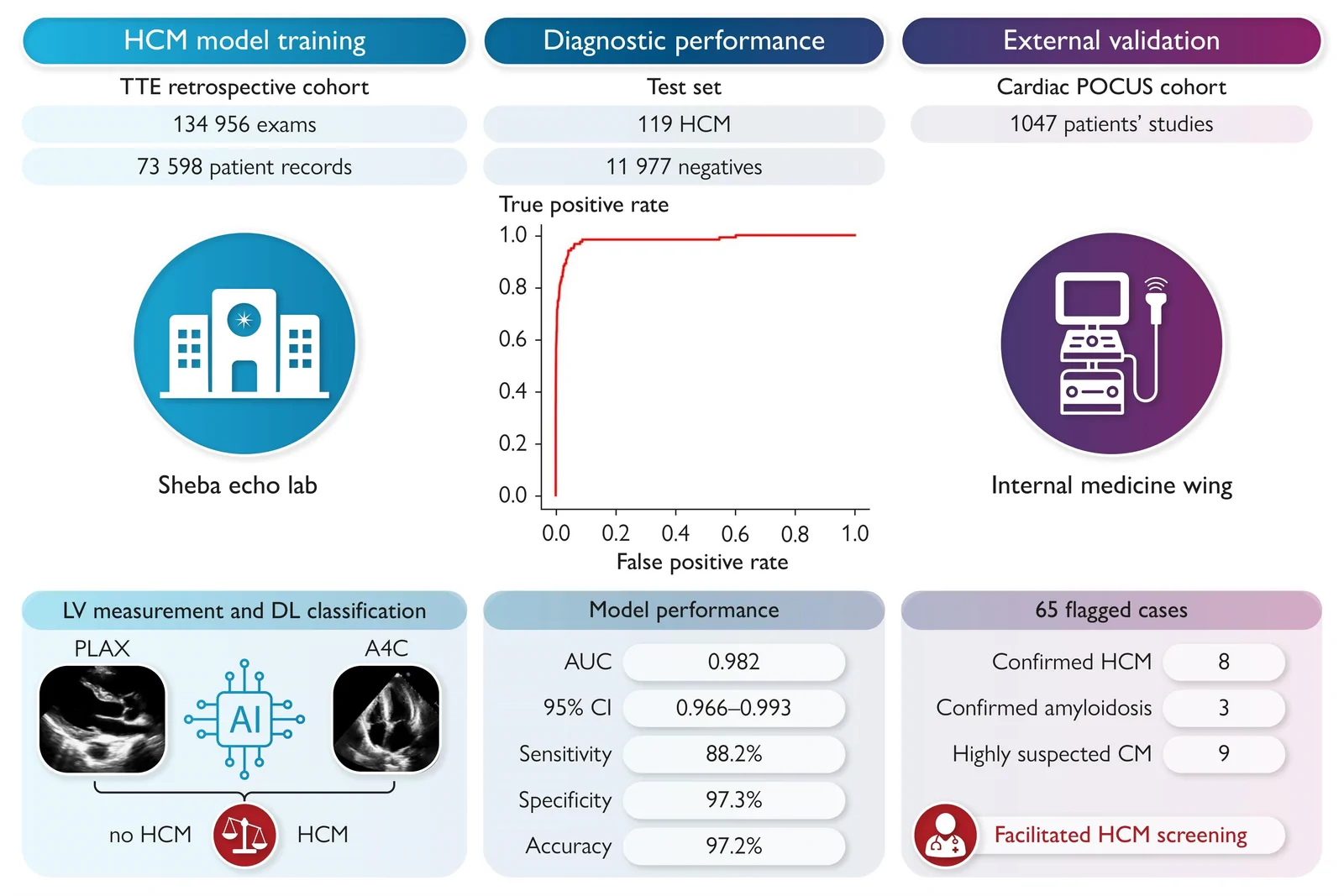

## Introduction

Hypertrophic cardiomyopathy (HCM) has a high estimated prevalence of 1:200–1:500. HCM is associated with significant morbidity and mortality from heart failure (HF), arrhythmias, and sudden cardiac death (SCD).^1,2^ Novel treatments have emerged, reducing morbidity and improving patients’ functional performance and quality of life.^2–4^ These advances underscore the importance of early recognition and treatment.

Unfortunately, only 10–20% of affected individuals are clinically recognized.^2,5^ Diagnosis is complicated by phenotypic variability and confounding overlapping conditions (athlete heart, hypertension, aortic stenosis). Furthermore, the clinical utility of echocardiography significantly depends on the skills and expertise of its users, from image acquisition through final interpretation. Thus, in cases where an echocardiographic reader is not minded towards HCM diagnosis, moderate left ventricular hypertrophy (LVH) may be missed or misdiagnosed.^6^ Finally, there is a great disparity in the availability of medical services among different populations, and access to echocardiography may be limited in underprivileged populations. Skilled staff to acquire and interpret cardiovascular imaging (CVI) studies are increasingly in short supply, further aggravating the problem of underdiagnosis, particularly in rural communities.^7^

Artificial intelligence (AI) has significantly developed in recent years. Novel machine deep learning (DL) techniques hold great promise to reshape CVI,^8,9^ both by enhancing diagnostic abilities and reaching otherwise remote and neglected patient populations.

Recognizing the unmet need of early HCM diagnosis, the purpose of the current study was to develop and validate a DL-echocardiographic model adaptable to point-of-care ultrasound (POCUS) for HCM screening using only two standard transthoracic echocardiographic (TTE) views.

## Methods

We used a large library of in- and outpatient expert, human-performed TTE studies to train and test the algorithm. Next, we retrospectively deployed the model on a cohort of patients hospitalized in the Internal Medicine Division who underwent cardiac POCUS.

### Study population and design

**Figure 1:**
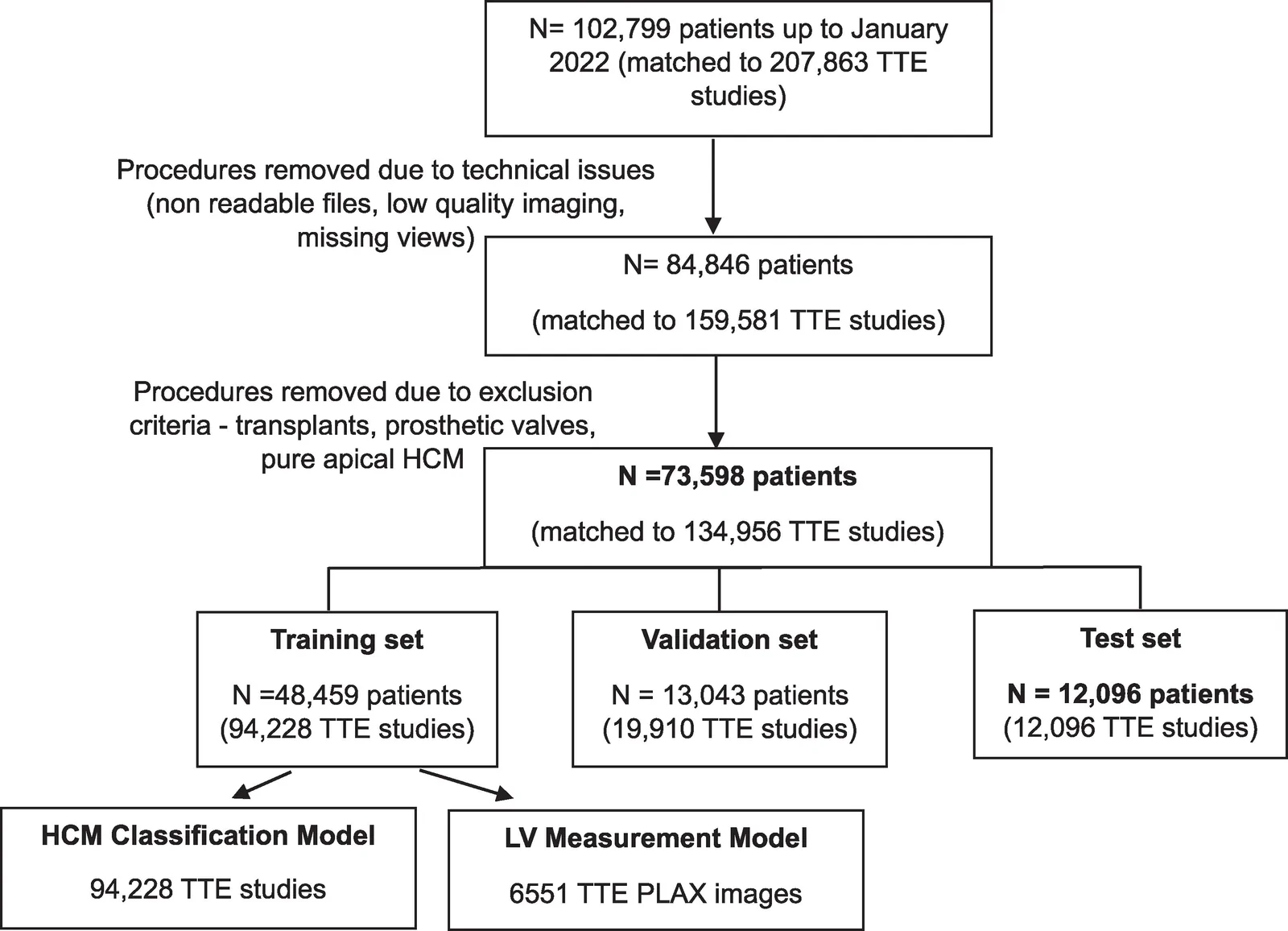
Study design. Cohort selection for model development and testing. From all available expert echocardiography studies, we first excluded studies for technical reasons (unreadable files, poor image quality, or failure of the automated view-classification model to detect the required A4C and PLAX views). We then applied clinical exclusion criteria (heart transplant, prosthetic valves, and apical HCM). The remaining studies were partitioned into training, validation, and test sets. The training set was used to train both the HCM classification model and the LV measurements model. Since LV measurements required manual annotation, only a subset of images from eligible exams contributed to this task. The validation set was used to set operating thresholds and assess the end-to-end pipeline. The test set comprised unique patients with one study per patient, yielding equal numbers of patients and studies. A4C, apical four chamber; PLAX, parasternal long axis; HCM, hypertrophic cardiomyopathy; LV, left ventricle.

This retrospective cohort included all adult patients (>18 years) who underwent formal standard expert TTE at Sheba Medical Center (SMC) (2007 to January 2022). Data from the SHEBAHEART big data registry were used. The initial cohort comprised 207 863 expert TTE studies linked to clinical data of 102 799 patients. Full TTE studies were carried out and interpreted by expert echocardiographers as per standard protocol.^10,11^ Examinations that did not pass the view classification and quality assessment process were excluded (see Supplementary material).^12^ TTEs of heart-transplanted patients and patients with prosthetic valves were excluded. The final cohort included 134 956 TTE studies matched to 73 598 unique patient records. Echocardiographic data were randomly divided into three mutually exclusive cohorts: train set (*n* = 94 228 expert TTE studies matched to 48 459 patients), validation set (*n* = 19 910 expert TTE studies matched to 13 043 patients) and test set (*n* = 12 096 expert TTE studies matched to 12 096 patients) (*Figure 1*).

The clinical record search for HCM-positive cases identified patients with ICD-matching codes (76% of cases) (hypertrophic cardiomyopathy; obstructive hypertrophic cardiomyopathy; other hypertrophic cardiomyopathy). Expert echocardiographic studies consistent with an HCM diagnosis and with LVH at any segment (73% of cases) were used to help identify patients with no previous diagnosis. Systolic anterior motion (SAM) and/or left ventricular outflow tract dynamic gradient/obstruction (LVOTO) were identified as supporting features (17% in the train/validation set and 31% in the test set). Additionally, 37% of HCM cases were confirmed by expert adjudication. Apical HCM was excluded based on TTE-confirmed diagnosis and ICD codes, as it is relatively uncommon (1–10% of HCM phenotypes) and is not reliably quantified on PLAX.

The study was approved by the SMC Institutional Review Board with a waiver of informed consent (SMC-21-8816). Strict maintenance of participants’ anonymity was ensured.

### Technology and model training

The DL model was developed by Aisap.ai, a private company, in collaboration with SMC. AISAP^®^ technology uses DL models for echocardiographic videos, is FDA-approved, and has extremely high accuracy in diagnosing reduced LV ejection fraction (LVEF) and valvular heart disease.^12^

### Algorithmic model development

**Figure 2:**
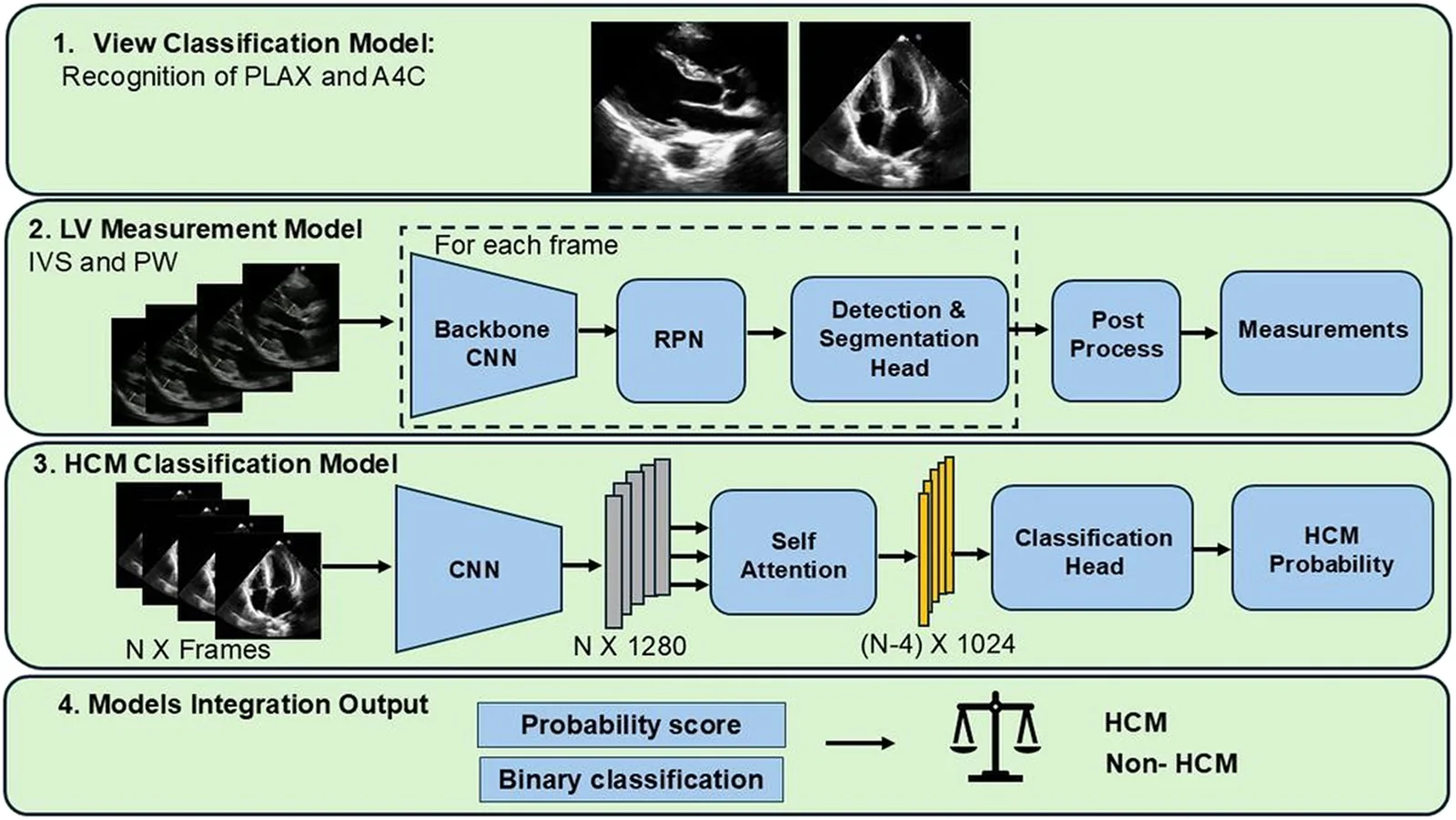
Illustration of algorithm development, integration, and final classification. A two-stage pipeline is shown: (1) View classification. The second stage involves two complementary models: (2) The LV structural measurement (segmentation) model applied to PLAX clips, extracting IVS and PW thickness at the level below the MV and at maximal thickness point. (3) The DL model captures spatial and temporal patterns of HCM (from A4C and PLAX clips). (4) The models are eventually integrated to yield HCM probability. HCM, hypertrophic cardiomyopathy; CNN, convolutional neural network; RPN, region proposal network.

We developed a two-stage pipeline designed to replicate expert workflow (*Figure 2*).

#### View classification model

The first stage identifies four echocardiographic views from raw ultrasound data: apical four-chamber (A4C), parasternal long axis (PLAX), parasternal short-axis at the aortic valve level, and subcostal view, as previously described.^12^ This gateway is essential for ensuring that only appropriate, high-quality clips are processed.^12^ The model architecture is based on EfficientNetV2.^13^

#### Left ventricular measurement model and hypertrophic cardiomyopathy classification model

The second stage combines two complementary models estimating HCM likelihood:

1. The LV structural measurement (segmentation) model applied to PLAX clips extracts the interventricular septum (IVS) and posterior wall (PW) thickness and maximal LV wall thickness (LVWT) (see Supplementary material online, *Figure S1*). The model performs two separate measurements for each anatomical structure at end-diastole. The first measurement, taken just below the level of the mitral valve leaflet tips, follows ASE guidelines.^10^ The second measures the maximal LVWT. The segmentation model is based on the Mask R-CNN FPN V2 architecture.^14^
2. The HCM classification model is a DL-based video classification that estimates disease probability using both PLAX and A4C clips. These clips serve as the input for a neural network that captures spatial and temporal patterns relevant to HCM (*Figure 2*). The architecture combines convolutional and transformer-based components. Feature extraction is performed using an EfficientNet backbone.^15^

Further details are provided in the Supplementary material.

#### Models integration

To generate a robust, clinically meaningful HCM prediction, we integrate outputs from the LV measurement (segmentation) and HCM classification models. This combines the binary output and probability score from the classifier model with IVS, PW, and maximal LVWT measurements from the segmentation model. The final decision-making logic operates on a threshold-based framework. When the classifier probability exceeds a high-confidence threshold, the case is labelled HCM. When it falls below a low threshold, the case is labelled non-HCM. For intermediate probabilities, we recalibrate the probability using the LV structural measurements (both basal IVS/PW and maximal LVWT from the segmentation model), adjusting the classifier score upward when maximal LVWT supports HCM morphology and downward otherwise. The final label is then assigned by comparing this recalibrated probability to the operating threshold.

### Model validation in an external cohort

The algorithm was retrospectively applied to a cohort of POCUS studies from all-comer hospitalized patients at the Internal Medicine Wing (May 2024–March 2025, institutional review board SMC-21-8816). Cardiac POCUS studies with four standard views were carried out by non-cardiologists (internal medicine residents) using Philips Lumify hand-held devices. Clinicians participated in a short POCUS training course as per pertinent guidelines and had no prior echocardiography experience. Because the AI algorithm depends on adequate image quality, users were guided during acquisition by real-time feedback on both technical quality and diagnostic adequacy, as previously reported^16,17^ (see Supplementary material). Patients were selected by the clinician based on clinical evaluation upon admission. We deployed the developed model to POCUS images of 1047 studies matched to 1047 patients (using two standard echocardiographic views). Results are expressed as a binary classification of screen-positive or negative, as explained earlier.

Cases classified by the model as screen-positive were subsequently examined by cardiologists who reviewed their medical records. True positive (TP) cases were adjudicated based on a pre-existing definite diagnosis (ICD codes), echocardiographic features from human-performed expert TTE consistent with HCM (LVH ≥1.3 cm, SAM, LVOT dynamic gradient), and other relevant clinical data (cardiac magnetic resonance (CMR), etc.). Medical records of cases flagged positive by the algorithm but without a prior HCM diagnosis were evaluated for relevant clinical and imaging characteristics (family history, SCD, ECG, expert TTE, CMR, etc.). Diagnosis was corroborated if the patient fulfilled the ESC guidelines criteria for HCM.^1^ Other positive cases were evaluated for a possible alternative diagnosis.

### Statistical analysis

We used R Software version 4.5.0 and Python Software. Continuous variables are presented as mean ± SD if normally distributed, or as median [interquartile range (IQR)] if skewed. Categorical variables are expressed as frequency (percentage). Student’s *t*-test was used to compare continuous data, while the *χ*^2^ test or Fisher’s exact test was used for categorical data comparison. *C* statistics were performed to analyse overall model performance. The 95% CIs were computed using 10 000 bootstrapped samples and by obtaining 95^th^ percentile ranges for each prediction. The operating point of the model was decided upon following examinations at varying thresholds (0.0 through 1.0), with corresponding values of sensitivity, specificity, positive predictive value (PPV), and negative predictive value (NPV) (see Supplementary material online, *Table S1*). To ensure a high sensitivity for HCM detection, while balancing TPs with false positives (FP), an operating cut-off of 0.24 was chosen. Subgroup analysis was carried out to evaluate model performance with respect to possible influencing variables. Here, 95% CI were computed using 2000 bootstrapped samples per subgroup. Delong’s test was used to report CI for area under the curve (AUC) values, and Wilson’s test was used to report CI for sensitivity and specificity comparisons.

## Results

### Test set patient clinical characteristics

Baseline characteristics of the test set are shown in *Table 1*. Overall, the majority of patients was male (57%). HCM-positive and negative patients were of similar age (median 74 [61, 86] vs. 75 [60, 85], respectively). Similar rates of hypertension (HTN), diabetes mellitus (DM), chronic kidney disease (CKD), and chronic obstructive pulmonary disease (COPD) were observed in both groups. Although the rate of ischaemic heart disease (IHD) did not differ between the groups, higher rates of percutaneous coronary interventions (PCI) were documented in HCM-negative patients (12.5% vs. 5.9%, *P* = 0.025). HCM-positive patients suffered from a higher rate of ventricular tachycardia (VT) (including non-sustained VT, 40.3% vs. 23.3%, *P* < 0.001). Concomitantly, significantly higher rates of ICD implantations were noted in this group (7.6% vs. 1.7%, *P* < 0.001). HCM-positive patients also had significantly higher rates of atrial fibrillation (AF) (35.3% vs. 16.6%, *P* < 0.001) and clinical HF (59.7% vs. 25.1%, *P* < 0.001). Higher rates of beta blockers (BB) (68.1% vs. 40%, *P* < 0.001) and mineralocorticoid receptor antagonists (MRAs) (18.5% vs. 8.3%, *P* < 0.001) were prescribed in the HCM-positive group.

**Table 1:** Baseline characteristics and expert-TTE derived parameters of the test set

Parameter	HCM negative	HCM positive	*P*-value	Overall
**Test cohort (*n*** **=** **12 096)**	11 977	119	NA	12 096
**Age (median [IQR])**	75 [60, 85]	73.5 [61, 86]	0.752	75 [60, 85]
<60 years, *n* (%)	3245 (27)	32 (27)	NA	
≥60 years, *n* (%)	8732 (73)	87 (73)	NA	
**Male, *n* (%)**	6798 (57.1)	72 (61)	0.455	6870 (57.2)
**HTN, *n* (%)**	3980 (33.2)	46 (38.7)	0.24	4026 (33.3)
**Dyslipidaemia, *n* (%)**	3080 (25.7)	37 (31.1)	0.206	3117 (25.8)
**DM, *n* (%)**	2253 (18.8)	15 (12.6)	0.098	2268 (18.8)
**CKD, *n* (%)**	718 (6.0)	6 (5.0)	0.846	724 (6.0)
**IHD, *n* (%)**	3103 (25.9)	27 (22.7)	0.463	3130 (25.9)
**PCI, *n* (%)**	1493 (12.5)	7 (5.9)	0.025	1500 (12.4)
**CABG, *n* (%)**	673 (5.6)	2 (1.7)	0.068	675 (5.6)
**Amyloidosis, *n* (%)**	31 (0.3)	2 (1.7)	0.042	33 (0.3)
**AF, *n* (%)**	1992 (16.6)	42 (35.3)	<0.001	2034 (16.8)
**COPD, *n* (%)**	459 (3.8)	6 (5.0)	0.467	465 (3.8)
**Heart failure, *n* (%)**	3012 (25.1)	71 (59.7)	<0.001	3083 (25.5)
**PAD, *n* (%)**	462 (3.9)	3 (2.5)	0.632	465 (3.8)
**Stroke, *n* (%)**	1387 (11.6)	13 (10.9)	1	1400 (11.6)
**VT,^a^ *n* (%)**	2791 (23.3)	48 (40.3)	<0.001	2839 (23.5)
**ICD, *n* (%)**	198 (1.7)	9 (7.6)	<0.001	207 (1.7)
**CRT, *n* (%)**	108 (0.9)	0	0.630	108 (0.9)
**Hb, g/dL (median [IQR])**	12.5 [10.9, 13.9]	13.07 [11.3, 14.6]	0.071	12.55 [10.9, 13.9]
**GFR, mL/min, (median [IQR])**	80 [58.8, 94.8]	71.88 [54.9, 92.2]	0.161	79.96 [58.8, 94.8]
**BMI (median [IQR])**	26.3 [23.5, 29.7]	28.34 [23.9, 32]	0.007	26.3 [23.5, 29.7]
**SBP, mmHg (median [IQR])**	129 [114, 144]	128 [114, 143.5]	0.823	129 [114, 144]
**DBP, mmHg (median [IQR])**	73 [65, 80]	71 [61, 80]	0.106	73 [65, 80]
**HR, b.p.m. (median [IQR])**	71 [63, 82]	69 [59.75, 78]	0.007	71 [63, 82]
**Beta blockers, *n* (%)**	4786 (40)	81 (68.1)	<0.001	4867 (40.2)
**MRA, *n* (%)**	998 (8.3)	22 (18.5)	<0.001	1020 (8.4)
**SGLT2 inhibitor, *n* (%)**	264 (2.2)	1 (0.8)	0.525	265 (2.2)
**Echocardiographic parameters from matched human performed expert TTE studies**				
**Visually estimated LVEF (%) (median [IQR])^b^**	60 [55, 60]	65 [60, 65]	<0.001	60 [55, 60]
**Expert TTE IVS (cm) (median [IQR])**	1.01 [0.9, 1.19]	1.67 [1.5, 2]	<0.001	1.01 [0.90, 1.19]
**Expert TTE IVS ≥ 1.3 cm, *n* (%)**	1008 (8.4)	119 (100)	<0.001	1127 (9.3)
**Expert TTE IVS ≥1.5 cm, *n* (%)**	336 (2.8)	106 (89.1)	<0.001	448 (3.7)
**Expert TTE IVS ≥ 1.8 cm, *n* (%)**	28 (0.2)	40 (33.6)	<0.001	68 (0.5)
**Expert TTE PW (cm) (median [IQR])**	0.9 [0.8, 1.0]	1.13 [1.0, 1.3]	<0.001	0.9 [0.80, 1.01]
**EDV (mL) (median [IQR])**	124 [95.2, 163]	82.84 [76.4, 89.26]	0.099	122.08 [94.1, 163]
**ESV (mL) (median [IQR])**	65 [42, 102]	11.61 [10.81, 12.42]	0.022	64 [41, 102]
**LA AP diameter (cm) (median [IQR])**	3.77 [3.4, 4.2]	4.30 [3.8, 4.8]	<0.001	3.8 [3.4, 4.2]
**LAVI (mL/m^2^) (median [IQR])**	29 [22, 38]	49.5 [36.75, 63.25]	<0.001	29 [22, 38]
**MR (moderate or above), *n* (%)**	704 (5.9)	10 (8.4)	0.3332	714 (5.9%)
**AS (moderate or above), *n* (%)**	478 (4)	6 (5)	0.728	484 (4.0%)
**TR (moderate or above), *n* (%)**	635 (5.3)	6 (5)	1	641 (5.3%)
**Estimated SPAP, mmHg (median [IQR])**	32 [27, 40]	37 [30.75, 43.25]	<0.001	32 [27, 40]

HTN, hypertension; DM, diabetes mellitus; CKD, chronic kidney disease; IHD, ischaemic heart disease; PCI, percutaneous coronary intervention; CABG, coronary artery bypass graft; AF, atrial fibrillation; COPD, chronic obstructive pulmonary disease; PAD, peripheral arterial disease; VT, ventricular tachycardia; ICD, implantable cardioverter defibrillator; CRT, chronotropic resynchronization therapy; Hb; Haemoglobin; GFR, glomerular filtration rate; BMI, body mass index; SBP, systolic blood pressure; DBP, diastolic blood pressure; MRA, mineralocorticoid receptor antagonist; LVEF, left ventricle ejection fraction; IVS, interventricular septum; PW, posterior wall; EDV, end-diastolic volume; ESV, end-systolic volume; LAAP diameter, left atrium anteroposterior diameter; LAVI, left atrium volume index; MR, mitral regurgitation; AS, aortic stenosis; TR, tricuspid regurgitation; SPAP, systolic pulmonary arterial pressure; HCM, hypertrophic cardiomyopathy.

^a^VT—all VT episodes, including NSVT, as documented on 24 h ECG Holter.

^b^Visually estimated LVEF extracted from expert TTEs of this historic cohort had high correlation with LVEF extracted from biplane Simpson.^11^

### Expert echocardiographic characteristics of the test set

Echocardiographic parameters from matched human-performed expert TTE studies were compared for true HCM-positive and HCM-negative patients (*Table 1*): LVEF was slightly higher in HCM-positive patients. The expert-based measurements of IVS and PW were significantly higher in HCM-positive patients, with a median thickness of 1.67 cm [IQR 1.5, 2] and of 1.1 cm [IQR 1.0, 1.3], respectively. When categorized into various expert TTE IVS thresholds, 89% of HCM patients had a septum thickness ≥1.5 cm, as opposed to only 3% of HCM-negative patients. HCM-negative patients with an expert TTE IVS ≥1.5 cm exhibited significantly higher rates of hypertension (HTN) (58%), aortic stenosis (18%), and amyloidosis (4%) compared to the rest of HCM-negative patients (see Supplementary material online, *Table S2*, all *P* < 0.05). Moreover, 34% of HCM-positive patients had an expert TTE IVS of more than 1.8 cm. The end-systolic volume was visibly lower in HCM patients (11.61 mL [10.81, 12.42] vs. 65 mL [42, 102], *P* < 0.05).

Consistent with higher rates of AF, the LA diameter and volume index were also significantly higher in HCM patients (LAVI 49.5 mL/m^2^ [36.75, 63.25] vs. 29 mL/m^2^ [22, 38]; *P* < 0.001). Out of 119 HCM patients, 37 (31%) had SAM and/or LVOTO on expert TTE.

When exploring intermediary outputs from the segmentation model component, the following IVS, PW, and maximal LVWT were measured (mean ± SD): 1.617 ± 0.320 cm, 0.903 ± 0.221 cm, and 1.872 ± 0.335 cm, respectively, for HCM-positive patients vs. 1.069 ± 0.250 cm, 0.771 ± 0.179, and 1.277 ± 0.271, respectively, for HCM-negative patients (see Supplementary material online, *Table S3*). Values are in agreement with those reported from matched human-measured expert TTE (*Table 1*) (see Supplementary material for detailed methodology).

Baseline characteristics of the validation set were similar to the test set (see Supplementary material online, *Tables S4* and *S5*).

### Hypertrophic cardiomyopathy model performance

**Figure 3:**
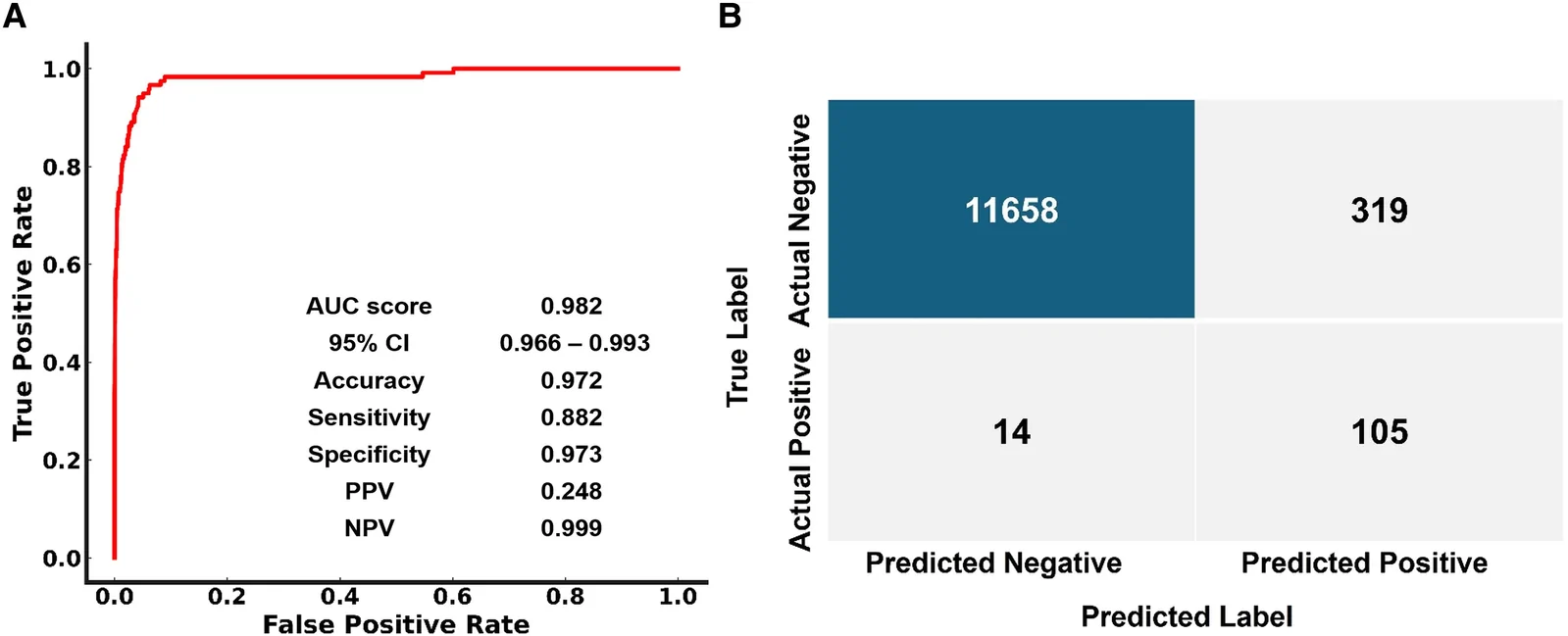
Model performance summary. (*A*) receiver operating curve of model performance with 95% CI. AUC indicates the area under the curve. PPV, positive predictive value; NPV, negative predictive value. (*B*) Confusion matrix indicating the actual HCM and predicted positive and negative cases.

Model performance summary of the test cohort is presented in *Figure 3*. The final model had an overall AUC of 0.982 (95% CI [0.966–0.993]), identifying HCM with a sensitivity of 88.2% and a specificity of 97.3%. The confusion matrix is presented in *Figure 3*, corresponding to a 24.8% PPV and a 99.9% NPV. When comparing false negative (FN) patients to TP, no difference in their clinical characteristics was noted. In matched expert TTE studies, TP patients had a slightly higher septal and PW thickness compared to 14 FN patients (IVS 1.7 cm [1.55–2] vs. 1.5 cm [1.49–1.6], *P* < 0.05). The ratio between IVS and PW was not different (see Supplementary material online, *Tables S6* and *S7*).

The LVH prevalence according to the DL-based classification (flagged as screen-positive vs. screen-negative) is provided in *Table 2*. It can be seen that 19% of the screen-negative patients (‘predicted negative’ in the confusion matrix) had LVH recognized by the segmentation model (model-measured maximal LVWT≥1.3 cm).

**Table 2:** LVH prevalence according to the DL-based classification in the test set

Group	*n*	^a^LVH positive	^a^LVH prevalence (%)
**Flagged as HCM (screen-positive)**	424	395	93.2
**Flagged as non-HCM** **(screen-negative)**	11 672	2257	19.3

DL, deep learning; HCM, hypertrophic cardiomyopathy; LVH, left ventricular hypertrophy.

^a^LVH is defined based on the maximal LV wall thickness threshold (≥1.3 cm), as measured by the intermediary step of the segmentation model.

### Subgroup performance analysis

Model sensitivity and specificity were analysed in different patient subgroups (*Table 3*). The model was only slightly more sensitive towards male patients (88.8% vs. 86.9%), but overall performance was similar in both genders (AUC of 0.984 vs. 0.979). The model had a better ability to predict HCM in patients younger than 60 years, with a higher sensitivity (96.8% vs. 88.1%).

**Table 3:** Subgroup analysis of model performance

HCM subgroup	AUC (95% CI)	Sensitivity % (95% CI)	Specificity % (95% CI)
**Gender**			
Male, (*n* = 72)	0.984 (0.969–0.99)	88.8% (79.6–94.3)	97.3% (96.9–97.6)
Female, (*n* = 47)	0.979 (0.953–1)	87% (74.3–93.9)	97.4% (96.9–97.8)
**Age**			
<60 years, (*n* = 32)	0.999 (0.997–1)	96.2% (81.1–99.3)	99.1% (98.7–99.4)
≥60 years, (*n* = 87)	0.976 (0.958–0.994)	85.9% (77.3–91.6)	96.8% (96.4–97.1)
**Expert TTE IVS thickness, cm**			
IVS ≥ 1.67 cm (*n* = 59)	0.895 (0.841–0.949)	94.9% (86.1–98.3)	57.5% (46.1–68.2)
IVS ≥ 1.5 cm (*n* = 106)	0.916 (0.884–0.946)	90.5% (83.5–94.8)	72.5% (67–76.6)
IVS <1.5 cm (*n* = 13)	0.948 (0.868–1)	69.2% (42.4–87.3)	98% (97.8–98.3)
**Expert TTE SPAP, mmHg**			
<40 mmHg (*n* = 59)	0.974 (0.948–1)	86.4% (75.5–93)	97.9% (97.6–98.2)
≥40 mmHg (*n* = 37)	0.982 (0.973–0.991)	86.5% (72–94.1)	95.2% (94.2–95.9)
**Heart failure**			
With HF (*n* = 71)	0.97 (0.946–0.994)	87.3% (77.6–93.2)	95% (94.2–95.7)
No HF (*n* = 48)	0.993 (0.988–0.997)	89.6% (77.8–95.5)	98.1% (97.8–98.4)
**Obesity**			
BMI <30 (*n* = 65)	0.985 (0.967–1)	87.7% (77.5–93.6)	97.7% (97.4–98)
BMI ≥30 (*n* = 44)	0.972 (0.946–0.998)	88.6% (76–95%)	96.2% (95.4–96.8)
**Co-existing AF**			
AF, (*n* = 42)	0.974 (0.946–1)	92.9% (81–97.5)	95.2% (94.2–96)
No AF, (*n* = 77)	0.984 (0.969–1)	85.7% (76.2–91.8)	97.7% (97.5–98)

The 95% CIs were computed using 2000 bootstrapped samples per subgroup.

Delong’s test was used to report CI for AUC values, and Wilson’s test was used to report CI for sensitivity and specificity comparisons.

AF, atrial fibrillation; AUC, area under the curve; BMI, body mass index; HCM, hypertrophic cardiomyopathy; HF, heart failure; IVS, interventricular septum; SPAP, systolic pulmonary arterial pressure; TTE, transthoracic echocardiograms

As to disease-related factors, the following results were found: IVS threshold increase from ≥1.5 to ≥1.67 cm (expert TTE-based median IVS) was associated with higher sensitivity (90.5% vs. 94.9%, respectively), albeit at the expense of lower specificity (72.5% and 57.5%, respectively). The model was also more sensitive with respect to HCM patients with AF (92.8% vs. 85.7%). No meaningful difference was observed in the subgroup analysis of obese patients, patients with pulmonary HTN, or with clinical HF.

### Point-of-care ultrasound cohort external validation

The HCM algorithm was retrospectively applied to POCUS studies of 1047 patients at the Internal Medicine Wing. Clinical and echocardiographic characteristics from prior matched expert TTEs of this cohort are presented in *Table 4*. Median age was 73 [61, 81] with 577 (55%) males. While the overall clinical profile of the POCUS cohort was similar to the test cohort, rates of comorbidities [HTN, COPD, CKD, DM, stroke, peripheral arterial disease (PAD), AF, etc.] were generally higher, representing a sicker population than that of allcomers at the Sheba Echo lab (*Table 1*). The HCM DL-model flagged 982 (93.8%) patients as screen-negative and 65 (6.2%) as screen-positive. Clinical characteristics of both groups were similar, with higher rates of AF and significantly higher BMI in screen-positive patients. Though not statistically significant, higher rates of BB and MRA use were noted in screen-positive patients, as shown for the test set (*Table 1*).

**Table 4:** Baseline clinical characteristics and parameters from matched expert TTE of POCUS cohort patients

Parameter	Overall POCUS	Flagged as screen-negative^a^	Flagged as screen-positive^a^	*P*-value
***n* (%)**	1047	982 (93.8)	65 (6.2)	NA
**Age (median [IQR])**	73 [61, 81]	73 [61, 81]	71 [63, 79]	0.449
**Male, *n* (%)**	577 (55.1)	541 (55.1)	36 (55.4)	1
**HTN, *n* (%)**	545 (52.1)	508 (51.7)	37 (56.9)	0.444
**Dyslipidaemia, *n* (%)**	481 (45.9)	447 (45.5)	34 (52.3)	0.306
**DM, *n* (%)**	312 (29.8)	295 (30)	17 (26.2)	0.577
**CKD, *n* (%)**	342 (32.7)	316 (32.2)	26 (40)	0.219
**IHD, *n* (%)**	341 (32.6)	317 (32.3)	24 (36.9)	0.494
**PCI, *n* (%)**	159 (15.2)	147 (15.0)	12 (18.5)	0.474
**CABG, *n* (%)**	67 (6.4)	61 (6.2)	6 (9.2)	0.299
**Amyloidosis, *n* (%)**	5 (0.48)	2 (0.2)	3 (4.6)	0.002
**AF, *n* (%)**	363 (34.7)	334 (34.0)	29 (44.6)	0.106
**COPD, *n* (%)**	80 (7.6)	74 (7.5)	6 (9.2)	0.627
**Heart failure, *n* (%)**	241 (23)	228 (23.2)	13 (20.0)	0.649
**PAD, *n* (%)**	66 (6.3)	62 (6.3)	4 (6.2)	1
**Stroke, *n* (%)**	149 (14.2)	137 (14.0)	12 (18.5)	0.357
**ICD, *n* (%)**	32 (3.1)	30 (3.1)	2 (3.1)	1
**CRT, *n* (%)**	17 (1.6)	16 (1.6)	1 (1.5)	1
**Hb, g/dL (median [IQR])**	12.7 [11.07, 14]	12.7 [11.04, 14]	12.7 [11.6, 14]	0.643
**GFR, mL/min, (median [IQR])**	79.5 [51.7, 94.6]	80 [52.3, 94.9]	73.1 [47.08, 91.5]	0.162
**BMI (median [IQR])**	26 [23.2, 29.6]	25.9 [23.1, 29.4]	28.3 [24.9, 31.7]	0.006
**SBP, mmHg (median [IQR])**	127 [112, 144]	127 [112, 144]	125 [117, 142]	0.684
**DBP, mmHg (median [IQR])**	71 [64, 80]	71 [63, 79]	73 [68, 81]	0.159
**HR, b.p.m. (median [IQR])**	73 [65, 85]	73 [65, 85]	73[65, 80]	0.668
**Beta blockers, *n* (%)**	574 (54.8)	532 (54.2)	42 (64.6)	0.122
**MRA, *n* (%)**	135 (12.9)	121 (12.3)	14 (21.5)	0.053
**SGLT2 inhibitor, *n* (%)**	278 (26.6)	260 (26.5)	18 (27.7)	0.885
**Echocardiographic parameters from matched expert TTE studies**				
**Formal expert TTE at Sheba, *n* (%)**	626 (59.7%)	577 (59%)	42 (64%)	
**Visually estimated EF, % (median [IQR])^b^**	60 [55–65]	60 [55–65]	60 [55–65]	0.89
**Expert TTE IVS (cm) (median [IQR])**	1.07 [0.9, 1.2]^*^	1.06 [0.9, 1.2]	1.26 [1.07, 1.46]	<0.001
**Expert TTE IVS ≥1.3 cm, *n* (%)**	79 (12.6)	61 (10.9%)	18 (43.9%)	<0.001
**Expert TTE IVS ≥ 1.5 cm, *n* (%)**	32 (5.1)	23 (4.1)	9 (22)	<0.001
**Expert TTE IVS ≥ 1.8 cm, *n* (%)**	7 (1.2)	2 (0.4)	5 (12.2)	<0.001
**Expert TTE PW (cm) (median [IQR])**	0.9 [0.8, 1.02]^*^	0.9 [0.8, 1]	1.09 [0.94, 1.2]	<0.001
**LA AP diameter (cm) (median [IQR])**	3.9 [3.5, 4.5]	3.9 [3.5, 4.5]	4.1 [3.6, 4.7]	0.150
**LAVI (mL/m^2^) (median [IQR])**	35 [25.75, 49]	35 [25, 48]	43.5 [29.25, 57.25]	0.073
**Estimated SPAP, mmHg (median [IQR])^c^**	36 [28, 46]	35.5 [28, 46]	38 [31.5, 46.5]	0.485
**Significant TR, *n* (%)^c^**	92 (16.4)	83 (15.8%)	9 (23.7%)	0.221
**Significant MR, *n* (%)^c^**	81 (15.2)	76 (15.4%)	5 (12.2)	0.411

BMI, body mass index; DM, diabetes mellitus; CKD, chronic kidney disease; GFR, glomerular filtration rate; HTN, hypertension; IHD, ischaemic heart disease; ICD, implantable cardioverter defibrillator; CRT, chronotropic resynchronization therapy; PCI, percutaneous coronary intervention; CABG, coronary artery bypass graft; AF, atrial fibrillation; COPD, chronic obstructive pulmonary disease; PAD, peripheral arterial disease; POCUS, point-of-care ultrasound; SBP, systolic blood pressure; DBP, diastolic blood pressure; MRA, mineralocorticoid receptor antagonist; MR, mitral regurgitation; PW, posterior wall; TR, tricuspid regurgitation; TTE, transthoracic echocardiograms

^a^Patients who were flagged as either screen-negative or positive by the model when the algorithm was deployed on their POCUS studies.

Amyloidosis cases denoted in the Table represent patients with confirmed diagnosis, as documented in their medical records. An additional amyloid case was found in the positively flagged group based on clinical data, though no ICD code was registered in the medical record.

^b^Visually estimated LVEF extracted from expert TTEs of this historic cohort had high correlation with LVEF extracted from biplane Simpson.^11^

^c^Only studies with these reported parameters were used for % calculations—MR, TR, and SPAP were reported only where a measurable Doppler was documented.

NA, not available.

^*^For comparison, in the total POCUS cohort, the mean ± SD IVS output derived from the intermediary segmentation model was 1.122 ± 0.247 cm, PW thickness was 0.879 ± 0.192 cm, and the maximal LVWT was 1.303 ± 0.270 cm. Mean measurements for confirmed positive and negative cases are not presented as the absolute number of expert-confirmed HCM patients is small (*n* = 8).

Out of 1047 patients in the POCUS cohort, 626 (59.7%) had undergone a prior formal expert TTE—577 (59%) of negatively flagged cases and 49 (75.4%) of positively flagged cases. Among patients with an available matched expert TTE, those flagged screen-positive by the DL-model had significantly greater expert-measured median septal thickness (1.26 [1.07, 1.46] vs. 1.06 [0.9, 1.2] cm; *P* < 0.001) and higher LAVI (43.5 [29.25, 57.25] vs. 35 [25, 48] mL/m^2^; *P* = 0.073) compared to patients classified as screen-negative. The percentage of patients with expert TTE-derived IVS ≥1.5 cm was 22% in the screen-positive group (vs. 4% in the negative group, *P* < 0.001), while 12% had an expert TTE IVS≥1.8 cm, compared to 0.4% in the screen-negative group (*P* < 0.001).

Extensive medical records review supervised by an HCM expert was performed for all 65 patients who were flagged as screen-positive by the algorithm. Medical history, comorbidities, ECG, and chronic drug therapy were available for all patients. Prior expert TTE studies were available for 49/65 positively flagged patients (75.4%), most performed in the last year. Following the review, out of 49 positively flagged patients with expert TTE, eight patients (8/49; 16%) were confirmed to have HCM, representing a prevalence of 0.76% in the entire POCUS cohort. Four out of eight definite cases exhibited LVOTO (50%), and three patients (37%) had SAM in the expert TTE. Additionally, three patients (3/49; 6%) had a definite diagnosis of cardiac amyloidosis. The remaining patients flagged as screen-positive did not undergo clinical evaluation in a specialized HF or cardiomyopathy clinic. However, medical records evaluation revealed there were at least nine additional cases (9/49, 18%) with characteristics highly suggestive of cardiomyopathy, particularly significant septal hypertrophy on expert TTE (IVS ≥1.5 cm in six out of nine patients), SAM (in three out of nine), LVOTO (in three out of nine) or pathological ECG (LVH or a pathological T wave pattern). The remaining patients did not have prior expert TTE for comparison and were lost to follow-up after the index hospitalization.

## Discussion

Overall, the algorithm demonstrated good performance with high accuracy, sensitivity, and specificity. HCM patients in the test set were of similar age to controls but had greater septal thickness and higher LAVI in expert TTE, consistent with prior reports.^18,19^ They also exhibited higher rates of HF and arrhythmias, predominantly AF, with increased VT and ICD implantation rates, as well as greater use of BB and MRA therapy, findings concordant with Vemulapalli *et al*.^19^

Subgroup analyses showed comparable performance in men and women, with stronger performance in patients <60 years. Indeed, HCM is among the leading causes of SCD among adolescents and young adults.^20^ HCM patients are generally younger than patients exhibiting LVH due to hypertensive heart, AS, or amyloidosis (50–60 years, vs. >65 years, respectively).^21–25^ The higher sensitivity of the model in younger patients is also consistent with previous publications: the CNN ECG model reported by Ko *et al.* performed particularly well in younger patients^26^; A similar trend was found by Hillis *et al.*^27^

Increased IVS thickness influenced sensitivity, at the expense of specificity; however, a careful interpretation is required due to the small number of HCM patients with expert-TTE IVS <1.5 cm in this set. Model robustness was preserved across subgroups (AUC >0.9; *Table 3*). External retrospective validation in a POCUS cohort demonstrated screening validity: among 1047 patients, 65 (6.2%) were classified as screen-positive, including eight confirmed HCM cases and three amyloidosis cases. The observed HCM prevalence (∼0.76%) was higher than that reported in the general population (0.5%)^2,5^ and higher than in POCUS cohorts reported by Oikonomou *et al*. (0.4–0.5%).^28^ Expert TTE-derived IVS thresholds (≥1.3, ≥1.5, and ≥1.8 cm) were markedly more prevalent in screen-positive patients, supporting biological plausibility. Despite higher comorbidity burden in the POCUS cohort, patterns distinguishing screen-positive from negative patients mirrored those in the test set (*Tables 1* and *4*), reinforcing model robustness.

In recent years, machine learning (ML) approaches have enabled generation of automated CVI modalities for heart disease diagnosis.^8,9,29,30^ While early echocardiographic DL focused on cardiac segmentation and functional assessment,^9,31–33^ applications are increasingly targeting disease prediction.^18,19,22–25,28,34–40^

Particular interest has been directed towards AI-assisted HCM screening, given underdiagnosis. Notable are several AI-enhanced electrocardiographic algorithms.^26,27,41–44^ ECG is widely available, rapid, low-cost and does not require specific operator training. Ko *et al.* reported a 12-lead ECG-based AI model able to detect HCM with a 0.95 AUC, 87% sensitivity and 90% specificity.^26^ Hillis *et al.* developed another ECG-based model with 0.975 AUC, 68.4% sensitivity, 99% specificity, and a PPV of 13.7%.^27^ Siontis *et al*. externally validated their model, achieving high performance (0.922 AUC, 87% diagnostic accuracy, 83% sensitivity, and 87% specificity).^41^ Our presented TTE-based model achieved comparable performance with a higher PPV. Rapid advances in handheld ultrasound technology and automated acquisition may progressively reduce historical barriers of cost and operator dependence associated with echocardiographic screening.^7^ Importantly, HCM diagnosis is imaging-defined; echocardiography directly interrogates structural phenotypes, whereas ECG relies on indirect electrophysiological correlates of disease. Furthermore, imaging has a clear advantage, providing invaluable structural information beyond diagnosis. For example, AI-enhanced CMR can approximate LGE without contrast injection.^45^ Moreover, frequent accurate assessment of LVEF and LVOT gradient is critical during myosin-inhibitor therapy titration and follow-up. Park *et al.* demonstrated DL-based prediction of severe LVOTO using PLAX views and showed treatment-related gradient reduction,^46^ highlighting how automated acquisition and analysis may facilitate novel cardiomyopathy therapies.^46,47^

Echocardiography remains a pillar of cardiac diagnosis.^34,46,47^ Several AI-based HCM detection models using retrospective TTE datasets have been described.^18,19,22,23,36^ Narula *et al*. combined speckle-tracking echocardiography-derived features with ML classifiers, achieving an AUC of 0.795 in a small cohort of 77 athlete hearts and 62 HCM cases.^22^ Zhang *et al.*^18^ trained convolutional neural networks (CNNs) on 14 035 echocardiograms, achieving an AUC of 0.93 for HCM detection and demonstrating associations between higher predicted disease probability and increased LA and LV mass.^18^ Duffy *et al*. employed a two-step AI strategy—LVH detection followed by CNN-based etiologic classification—achieving AUCs of 0.79 for amyloidosis and 0.89 for HCM in an external validation set.^36^ These approaches, mostly based on single or two echocardiographic views^22,23,32,36^ are conceptually aligned with our two-view hybrid model.

Notably, these prior models relied on expert-acquired echocardiograms mostly from academic centres.^18,19,22,36^ However, low-cost screening tools that can be implemented and interpreted with relative ease by non-cardiologists are sought. The rising popularity of portable ultrasound devices highlights the need for simplified and scalable models applicable to videos of variable quality. Thus, our model, comprising both structural and spatiotemporal features, was developed using only two echocardiographic views. This abbreviated approach using PLAX and A4C has proven successful in our previous works.^12,16^ Recently, PanEcho, an automated AI system, performed multiple echocardiographic diagnostic classification tasks, showing good performance even in limited POCUS protocols.^48^ The US Food and Drug Administration has granted approval for several AI-backed software programs enabling novice acquisition of diagnostic-quality echocardiograms.^7,49^ Yet, POCUS-implemented HCM models remain scarce. Oikonomou and colleagues^28^ developed a single-view POCUS-adapted CNN achieving AUCs of 0.89–0.903 for HCM.^28^ Prevalence of HCM in their POCUS cohorts was 0.4–0.5%. Their model benefits from customized TTE training emphasizing low-quality acquisitions and off-axis, non-traditional views.^28^ In contrast, our model, trained on expert TTE acquisitions, excluded poor-quality studies. Yet, it successfully identified HCM cases when applied to POCUS, yielding a higher observed prevalence. In that sense, our work is innovative.

### Study strengths and limitations

Sheba is the largest tertiary medical centre in Israel with a major referral cardiomyopathy clinic, supported by The SHEBAHEART big data registry (22 000 TTE studies per year). This allows model development on a large, well-annotated dataset.

Our algorithm integrates structural measurements with learned temporal patterns in cardiac motion. This hybrid strategy leverages the strengths of the data-driven pattern recognition classification and the anatomical specificity of the measurement model, improving robustness, interpretability, and mitigation of borderline predictions.

The POCUS cohort is of paramount significance, demonstrating validity in unselected patients scanned bedside by novices with hand-held devices. Scans were performed at the treating physician’s discretion, minimizing selection bias. Consistent phenotypic patterns across test and POCUS cohorts support generalizability. Among 65 positively flagged patients, 49 (75%) had expert TTE, ECG, and comorbidities data. Of the 49 patients with expert TTE and full data, 8 (16%) had confirmed HCM, 3 (6%) amyloidosis, and 9 (18%) had high suspicion for cardiomyopathy based on ECG and TTE. The remaining POCUS-positive patients did not undergo a comprehensive cardiomyopathy evaluation. If it were to be done, additional HCM cases may have been confirmed. This will be addressed in our future work. Interestingly, high AI-defined probabilities have been associated with worse long-term outcomes, even among those who are never diagnosed with cardiomyopathy.^28^

Several drawbacks merit consideration: first, this retrospective study lacks multi-centre validation. Referral bias of clinically more severe HCM to Sheba’s specialty clinics is possible. Yet, the model did recognize definite HCM cases in an all-comers POCUS cohort undergoing ‘opportunistic’ scans by non-cardiologists. Second, training included patients with repeated echocardiographic examinations, thus temporal changes (e.g. early absence of LVH or late-stage re-modelling) could impose diagnostic challenges. However, inclusion of HCM-positive cases in the training process required the presence of LVH on expert TTE, ensuring that the model learns from images reflecting the disease phenotype. Third, 89% of HCM test set patients had an IVS ≥1.5 cm on expert TTE, while in reality, some HCM patients may have only borderline hypertrophy. Nevertheless, subgroup analysis suggests reasonable sensitivity even below this threshold. Furthermore, the segmentation-model recognized LVH in 19% of the screen-negative patients in the test set, reinforcing that LVH is not the sole operating criteria of the model. Fourth, PPV was modest (25%), similar to results reported in other HCM screening models.^27,28,37^ This could be a reflection of the models’ ability to balance false positive cases with TPs. Screening tests inherently suffer from low specificity due to a trade-off with high sensitivity. To ensure high sensitivity for HCM detection, a PPV of 25% was satisfactory here. Since the current model is meant for screening and not as a definite diagnostic tool, further subsequent evaluation will be required for positively flagged patients. Furthermore, a higher PPV is expected when the model is applied in a population with a high pre-test probability.

Fifth, the model does not explicitly distinguish HCM from amyloidosis, leading to some overlap, but effectively identifies high-risk LVH patients, appropriate for screening. Importantly, AI-enhanced screening, however promising, does not replace comprehensive clinical assessment. The differential diagnosis of LVH can be particularly challenging in real-life settings, where the phenotypic presentation of HCM is variable and concurrent comorbidities may contribute to LVH and confound diagnosis. In clinical practice, multimodal imaging approaches, genetic testing and even tissue biopsy are often required before a final diagnosis can be made.^1,50^

Finally, our model was trained on expert TTEs, and though POCUS-deployed results are encouraging, generalizability and statistical power are limited by sample size (only eight confirmed cases by expert adjudication). Nevertheless, the HCM prevalence of 0.76% in this POCUS cohort is still higher than that reported in the general population (0.2–0.5%). Larger retrospective and prospective POCUS validations are underway.

## Conclusions

We have developed an echocardiographic-based DL algorithm able to identify HCM with high sensitivity and specificity. We provide a proof-of-concept on how this screening model can be easily adapted to POCUS carried out by non-cardiologists. It is our hope that further development of such models applicable to POCUS will promote equity in cardiac care, facilitating early disease screening even in the most resource-deprived populations.

## Supplementary Material

ztag140_Supplementary_Data

## Data Availability

The Digital Imaging and Communications in Medicine videos are housed at the Sheba Medical Center Hospital Echocardiography core laboratory. Third-party contractual agreements prohibit sharing these videos publicly. Assessment of the original videos can be made on site at Sheba Medical Center by request to the corresponding author and with appropriate data-use agreements. The code used in the software is not publicly available, as it is considered proprietary intellectual property by the sponsor. The software will be made available by request to the sponsor (aisap.ai) for the purposes of reproducing the presented results.

## Lead author biography

Nour Karra, MD, PhD, is a cardiology fellow at the Heart and Vascular Center, Sheba Medical Center. She earned her PhD in Pharmaceutics from the Hebrew University of Jerusalem, where she focused on targeted nanomedicine and translational therapeutics, and subsequently earned her MD from Tel-Aviv University. Her current research integrates artificial intelligence with cardiovascular imaging. With a background bridging molecular therapeutics and real-world cardiovascular practice, her work focuses on implementing precision technologies to improve diagnostic equity and clinical outcomes. She is actively involved in clinical research, innovation programmes, and academic teaching within a high-volume tertiary cardiovascular centre.
