# Supplementary material for: Point-of-care echocardiography screening for hypertrophic cardiomyopathy using automated deep-learning analysis
Source: Eur Heart J Digit Health. 2026 Sep 9;7(8):ztag140. doi: 10.1093/ehjdh/ztag140 (PMC13556451; doi:10.1093/ehjdh/ztag140)
Supplement: Supplementary file 1 [file ztag140_supplementary_data.doc]

***1. Algorithmic Model Development***

***1.1. View Classification Model***

To ensure accurate input to the diagnostic pipeline, we developed a DL-based view classification model trained to recognize 18 TTE views, as previously described.^12^ The model architecture is based on EfficientNetV2^11^ and was trained using 11,000 manually labelled ultrasound video clips. Validation was performed on a separate dataset of 3,000 frames, each labelled by two certified echocardiography technicians.

The training process used a standard cross-entropy loss function and incorporated optimization strategies to prioritize performance on four primary diagnostic views: A4C, PLAX, parasternal short-axis at the aortic valve level (PSAX AV), and subcostal (SC). To enhance generalizability, we applied a range of augmentations including geometric transformations (rotation, translation, scaling), photometric shifts (brightness, contrast, gamma), and ultrasound-specific artifacts (shadowing, attenuation, resolution variation, and sharpening). These augmentations simulated variability across different acquisition devices and imaging conditions. Although trained to distinguish between 18 view classes, the model's outputs were later grouped into 7 categories used for inference: A4C, PLAX, PSAX AV, subcostal IVC (SC IVC), apical five-chamber (A5C), apical three-chamber (A3C), and apical two-chamber (A2C). All other views were consolidated into a general, "none of the above" category.

The model operates at the frame level, assigning view probabilities to each frame in a clip. View classification for a given clip is then determined by aggregating frame-level predictions across the sequence. Only loops with a predicted view confidence above a predefined threshold were passed to the HCM prediction models.^12^ Clips that did not meet this threshold were excluded.^12^

The model was evaluated on a dedicated validation set of 500 procedures and achieved an overall classification accuracy of 98% for the 4 main views.

***1.2. LV Measurement Model***

The measurement model was developed to extract clinically relevant structural parameters of the interventricular septum (IVS) and posterior wall (PW) from PLAX echocardiographic clips (Figure S1). The model performs two separate measurements for each anatomical structure at end-diastole. The first measurement, taken just below the level of the mitral valve leaflet tips, follows ASE guidelines.^10^ The second measures the maximal wall thickness. We intentionally did not apply a strict threshold-based rule (>15 mm) across all predictions for several reasons: First, as with any automated measurement system, there may be variability or occasional inaccuracies, including underestimation of maximal thickness in certain frames or segments. Second, increased wall thickness is not specific and may also be observed in hypertension or aortic stenosis, particularly in cases of concentric hypertrophy. Finally, for high- and low-confidence predictions, the classification model already provides robust discrimination, and enforcing a hard threshold could reduce overall performance.

The segmentation model is based on the Mask R-CNN FPN V2 architecture,^12^ which combines object detection and instance segmentation. The implementation utilizes a ResNet-50 backbone coupled with a Feature Pyramid Network (FPN) for multi-scale feature extraction.

The model was optimized using stochastic gradient descent (SGD). Training was performed using standard Mask R-CNN optimization techniques, including a multi-task loss composed of classification loss, bounding box regression loss, and mask loss.

For each input PLAX video, the model generates pixel-level segmentation masks of the relevant structures across all frames. These structures include the left ventricle, right ventricle, aorta, left atrium, IVS and PW. A post-processing pipeline then identifies the end-diastolic frame, from which measurements are extracted using the segmentation masks. Pixel distances are converted into real-world length units using metadata extracted from the DICOM headers. On an internal test set, the model achieved a mean absolute error (MAE) of 1.3 mm for IVS measurements and 1.2 mm for PW measurements, demonstrating strong agreement with expert manual measurements.

***1.3. HCM Classification Model***

The classification model is used to estimate the probability of HCM from echocardiographic video data using a deep learning-based framework. The model receives as input a combination of PLAX and A4C clips that pass a predefined confidence threshold determined by the view classification model. These clips serve as the input for a neural network architecture that captures both spatial and temporal patterns relevant to HCM.

The architecture of the model combines convolutional and transformer-based components. Feature extraction from each input frame is performed using an EfficientNet backbone,^13^ pretrained on the view classification task to ensure echocardiography domain relevance. The resulting feature vectors from each frame are passed into a multi-head self-attention module, which integrates information across time steps by attending to both spatial and temporal dependencies. The aggregated output is then processed through a max-pooling operation and fed into a multi-layer perceptron (MLP) with a hidden layer and an output layer for binary classification, HCM-positive or negative.

From each PLAX and A4C loop, 10 frames are sampled within a 1.5-second window to ensure adequate coverage of the cardiac cycle. These frames are concatenated together so that the model receives them as a combined input sequence.

Training was performed using a standard cross-entropy loss function. In addition to the augmentations used for the view classification model, we leveraged the presence of multiple valid PLAX and A4C loops available per study. During training, different loop combinations were sampled in each epoch to improve generalization and robustness. This strategy, coupled with variable frame sampling, served as an effective form of data augmentation.

During inference, the model evaluates multiple loop combinations when more than one valid clip is available per view. The final HCM probability is obtained by averaging the output scores across all combinations, resulting in a stable and reliable diagnostic prediction.

**Supplementary Figure S1: IVS and PW measurement examples in the segmentation model at end diastole in PLAX**

***2. POCUS external validation***

Because the AI algorithm depends on adequate image quality, users were guided during acquisition by real-time feedback on both technical quality and diagnostic adequacy. This approach has been already applied in our groups previous works.^16,17^

The AI-powered software integrated into the ultrasound system assessed the diagnostic quality of each video clip. This capability provided real-time feedback on image quality and operator performance, allowing immediate technique correction and enabling diagnostic-quality imaging after limited training. Before saving each clip, the system displayed a numerical quality score (as a percentage), enabling operators to adjust the probe position and consistently obtain AI-interpretable images (i.e., clips that yield an AI-generated output). (see Supplementary Figure S2). Notably, each resident's first twenty scans were supervised by a cardiologist before independent scanning. ^16, 17^

**Supplementary figure S2: Examples of diagnostic value indicators (adapted from Fisher *et al.* 2026, Mayo Clin Proc Digit Health.)^17^:** the indicator in the upper left corner of each figure provides real-time feedback on the diagnostic quality, including proper plane position and image quality. A grading of the image is provided on a scale of 0-100% (non-diagnostic to fully diagnostic) and also by a colour scale visualisation (red- poor quality, orange- medium quality, green- good quality). Provided are examples of PLAX images of varying acquisition quality.

**Statistical analysis**

**Supplementary Table S1: Operating points of the model at thresholds 0.0 through 1.0, showing sensitivity, specificity, PPV, and NPV.**

| **Cutoff** | **Sensitivity** | **Specificity** | **PPV** | **NPV** |
| --- | --- | --- | --- | --- |
| **0.20** | 0.924 | 0.958 | 0.181 | 0.9992 |
| **0.25** | 0.882 | 0.973 | 0.248 | 0.9988 |
| **0.30** | 0.840 | 0.979 | 0.281 | 0.9984 |

**Results - Saliency maps**

To enhance the model’s explainability, sample saliency maps were generated for the most representative cases using gradient-weighted class activation mapping. Representative Gradient-weighted Class Activation Mapping saliency maps are shown for the most confident true positive predictions of HCM (Supplementary Figure S2). An intense signal is evident in A4C and PLAX views at the IVS and basal septum.

**Supplementary Figure S3:**  **Saliency maps.** Gradient-weighted Class Activation Mapping saliency are shown for HCM in A4C (A) and PLAX (B) views. The colour range denotes areas of importance from blue to red

**Supplementary Table S2: Non-HCM test set subgroup with expert TTE IVS ≥ 1.5 cm**

| **Parameter** | **HCM negative with IVS ≥1.5 cm** | | **All HCM-negative** | |
| --- | --- | --- | --- | --- |
| **N (% from HCM negative group)** | 336 (2.8) | 11,977 | |  |
| **HTN, N (%)** | 197 (58.6) | 3980 (33.2) | |  |
| **Dyslipidaemia, N (%)** | 126 (37.5) | 3080 (25.7) | |  |
| **Diabetes, N (%)** | 103 (30.7) | 2253 (18.8) | |  |
| **CKD, N (%)** | 54 (16.1) | 718 (6.0) | |  |
| **IHD, N (%)** | 132 (39.3) | 3103 (25.9) | |  |
| **PCI, N (%)** | 59 (17.6) | 1493 (12.5) | |  |
| **CABG, N (%)** | 25 (7.4) | 673 (5.6) | |  |
| **Amyloidosis, N (%)** | 14 (4.2) | 31 (0.3) | |  |
| **AF, N (%)** | 119 (35.4) | 1992 (16.6) | |  |
| **COPD, N (%)** | 21 (6.2) | 459 (3.8) | |  |
| **Heart Failure, N (%)** | 180 (53.6) | 3012 (25.1) | |  |
| **PAD, N (%)** | 27 (8) | 462 (3.9) | |  |
| **Stroke, N (%)** | 65 (19.3) | 1387 (11.6) | |  |
| **VT, N (%)** | 140 (41.7) | 2791 (23.3) | |  |
| **CRT, N (%)** | 7 (2.1) | 108 (0.9) | |  |
| **Hb, g/dl (median [IQR])** | 12.25 [10.79, 13.84] | 12.5 [10.9, 13.9] | |  |
| **GFR, ml/min, (median [IQR])** | 60.50 [40.79, 79.51] | 80 [58.8, 94.8] | |  |
| **BMI (median [IQR])** | 28.36 [25.32, 32.45] | 26.3 [23.5, 29.7] | |  |
| **SBP, mmHg (median [IQR])** | 141 [122.25, 155] | 129 [114, 144] | |  |
| **DBP, mmHg (median [IQR])** | 72 [64, 81] | 73 [65, 80] | |  |
| **HR, bpm (median [IQR])** | 71 [61, 81] | 71 [63, 82] | |  |
| **Beta Blockers, N (%)** | 212 (63.1) | 4786 (40) | |  |
| **MRA, N (%)** | 55 (16.4) | 998 (8.3) | |  |
| **SGLT2 Inhibitor, N (%)** | 10 (3.0) | 264 (2.2) | |  |
| **Echocardiographic parameters from matched expert TTE studies** | | | |  |
| **Visually estimated LVEF (%) (median [IQR])*^a^*** | 60 [50, 60] | 60 [55, 60] | |  |
| **Expert TTE IVS (cm) (median [IQR])** | 1.54 [1.5, 1.64] | 1.01 [0.9, 1.19] | |  |
| **Expert TTE PW (cm) (median [IQR])** | 1.12 [1, 1.3] | 0.9 [0.8, 1.0] | |  |
| **EDV (ml) (median [IQR])** | 110 [99, 194.5] | 124 [95.2, 163] | |  |
| **ESV (ml) (median [IQR])** | 67 [43, 145] | 65 [42, 102] | |  |
| **LA AP Diameter (cm) (median [IQR])** | 4.3 [3.9, 4.72] | 3.77 [3.4, 4.2] | |  |
| **LAVI (ml/m^2^) (median [IQR])** | 38 [30.75, 49] | 29 [22, 38] | |  |
| **MR (moderate or above), N (%)** | 39 (11.6) | 704 (5.9) | |  |
| **AS (moderate or above), N (%)** | 63 (18) | 478 (4) | |  |

In χ² testing for the categorical variables, all P< 0.05 (except CABG and SGLT2-I)

**Supplementary Table S3 – AI-derived mean IVS, PW and maximal wall thickness measurements taken from the intermediary segmentation component of the DL-model for the Test set**

| **Group** | **PW Mean ± SD, cm** | **IVS Mean ± SD, cm** | **Max LVWT, Mean ± SD, cm** |
| --- | --- | --- | --- |
| **Overall Test set** | 0.772 ± 0.180 | 1.074 ± 0.257 | 1.283 ± 0.278 |
| **True HCM-negative** | 0.771 ± 0.179 | 1.069 ± 0.250 | 1.277 ± 0.271 |
| **True HCM positive** | 0.903 ± 0.221 | 1.617 ± 0.320 | 1.872 ± 0.335 |

AI measurements of IVS and PW thickness from the segmentation model are in agreement with the parallel measurements derived from matched human, expert-TTE reported in Table 1.

**Supplementary Table S4: Baseline characteristics of the training and validation set**

| **Parameter** | **HCM-negative** | **HCM-positive** | **p Value** |
| --- | --- | --- | --- |
| **Training and validation (N=61,502)** | 60,732 | 770 |  |
| **Age (median [IQR])** | 65 [52, 76] | 68 [58, 78] | <0.001 |
| **Male, N (%)** | 34479 (57.2) | 460 (59.8) | 0.1529 |
| **HTN, N (%)** | 18909 (31.1) | 312 (40.5) | <0.001 |
| **Dyslipidaemia, N (%)** | 13923 (22.9) | 246 (31.9) | <0.001 |
| **Diabetes, N (%)** | 10822 (17.8) | 102 (13.2) | 0.0011 |
| **CKD, N (%)** | 11203 (18.4) | 177 (23.0) | 0.0015 |
| **IHD, N (%)** | 15110 (24.9) | 162 (21) | 0.016 |
| **PCI, N (%)** | 6841 (11.3) | 70 (9.1) | 0.0657 |
| **CABG, N (%)** | 2965 (4.9) | 12 (1.6) | <0.001 |
| **Amyloidosis, N (%)** | 156 (0.3) | 14 (1.8) | <0.001 |
| **AF, N (%)** | 9159 (15.1) | 196 (25.5) | <0.001 |
| **COPD, N (%)** | 2252 (3.7) | 37 (4.8) | 0.1330 |
| **Heart Failure, N (%)** | 16472 (27.1) | 504 (65.5) | <0.001 |
| **PAD, N (%)** | 2151 (3.5) | 28 (3.6) | 0.9657 |
| **Stroke, N (%)** | 6335 (10.4) | 70 (9.1) | 0.2499 |
| **ICD, N (%)** | 2179 (3.6) | 100 (13.0) | <0.001 |
| **CRT, N (%)** | 1264 (2.1) | 37 (4.8) | <0.001 |
| **Hb, g/dl (median [IQR])** | 12.6 [10.99, 14] | 12.78 [11.12, 14.1] | 0.1341 |
| **GFR, ml/min, (median [IQR])** | 83.46 [61.7, 99.34] | 74.58 [52.9, 94.06] | <0.001 |
| **BMI (median [IQR])** | 26.54 [23.74, 30.1] | 27.36 [24.6, 30.92] | <0.001 |
| **SBP, mmHg (median [IQR])** | 130 [115, 144] | 130 [116, 146] | 0.0299 |
| **DBP, mmHg (median [IQR])** | 73 [65, 81] | 72 [63, 80] | 0.081 |
| **HR, bpm (median [IQR])** | 72 [63, 83] | 69 [60, 79] | <0.001 |
| **Beta Blockers, N (%)** | 24724 (40.7) | 497 (64.5) | <0.001 |
| **MRA, N (%)** | 4689 (7.7) | 97 (12.6) | <0.001 |
| **SGLT2 Inhibitor, N (%)** | 1039 (1.7) | 9 (1.2) | 0.3103 |

**Supplementary Table S5: Expert TTE derived e**chocardiographic characteristics of training and validation set

| **Parameter** | **HCM-negative** | **HCM-positive** | **p Value** |
| --- | --- | --- | --- |
| **Training and validation (N=61,502)** | 60,732 | 770 |  |
| Visually estimated LVEF (%) (median [IQR]) | 60 [55, 60] | 65 [60, 65] | <0.001 |
| Expert TTE IVS (cm) (median [IQR]) | 1.02 [0.90, 1.2] | 1.63 [1.50, 1.88] | <0.001 |
| Expert TTE IVS≥1.3 cm (%) | 5543 (9.2) | 719 (93.3) | <0.001 |
| Expert TTE IVS ≥ 1.5 cm (%) | 2025 (3.4) | 656 (89.6) | <0.001 |
| Expert TTE IVS ≥ 1.8 cm (%) | 282 (0.5) | 195 (26.6) | <0.001 |
| Expert TTE PW (cm) (median [IQR]) | 0.92 [0.80, 1.02] | 1.13 [1.00, 1.30] | <0.001 |
| End Diastolic Volume (ml) (median [IQR]) | 126 [96.21, 167] | 137.5 [72.75, 155.5] | 0.4595 |
| End Systolic Volume (ml) (median [IQR]) | 66 [30.82, 110.25] | 60 [32.75, 105.75] | 0.8636 |
| LA AP Diameter (cm) (median [IQR]) | 3.8 [3.4, 4.2] | 4.3 [3.9, 4.7] | <0.001 |
| LAVI (ml/m^2^) (median [IQR]) | 28 [22, 38] | 41 [32, 54] | <0.001 |
| TR (moderate or above), N (%) | 2856 (4.7%) | 38 (4.9%) | 0.8282 |
| Estimated SPAP, mmHg (median [IQR]) | 32 [27, 40] | 36 [31, 45] | <0.001 |

Out of 770 positive cases in this set, 131 patients (17%) had SAM and /or LVOTO documented on TTE.

**Supplementary Table S6: Clinical characteristics of false negative and true positive HCM patients in the test set**

| **Variable** | **False negative** | **True positive** | **p_value** |
| --- | --- | --- | --- |
| N | 14 | 105 |  |
| Probability score (numeric) (model prediction) | 0.18 [0.1–0.2] | 0.85 [0.65–0.95] | <0.001 |
| Age, (median [IQR]) | 78 [72–90.75] | 72.5 [60–85.25] | 0.263 |
| BMI (median [IQR]) | 28.3 [25.34–30.79] | 28.53 [23.91–32.1] | 1 |
| HR, bpm (median [IQR]) | 69 [60.5–74] | 69 [59–79] | 0.796 |
| SBP, mmHg, (median [IQR]) | 137 [124–142] | 126.5 [114–143.75] | 0.338 |
| DBP, mmHg, (median [IQR]) | 70 [60–76] | 71 [61.25–80] | 0.682 |
| Male, N (%) | 8 (57.1%) | 64 (61.5%) | 0.752 |
| Asymmetric septal pattern | 12 (85.7%) | 72 (68.6%) | 0.228 |
| HTN, N (%) | 7 (50%) | 39 (37.1%) | 0.353 |
| DM, N (%) | 2 (14.3%) | 13 (12.4%) | 0.69 |
| IHD, N (%) | 3 (21.4%) | 24 (22.9%) | 1 |
| MI, N (%) | 2 (14.3%) | 13 (12.4%) | 0.69 |
| AF, N (%) | 3 (21.4%) | 39 (37.1%) | 0.374 |
| COPD, N (%) | 1 (7.1%) | 5 (4.8%) | 0.536 |
| CKD, N (%) | 2 (14.3%) | 4 (3.8%) | 0.147 |
| CVA, N (%) | 2 (14.3%) | 11 (10.5%) | 0.65 |
| PAD. N (%) | 0 (0.0%) | 3 (2.9%) | 1 |
| CABG, N (%) | 0 (0.0%) | 2 (1.9%) | 1 |
| PCI, N (%) | 2 (14.3%) | 5 (4.8%) | 0.191 |

**Supplementary Table S7: Expert TTE derived echocardiographic characteristics of false negative and true positive HCM patients in the test set**

| **Variable** **False negative** \| **True positive** \| **p_value** \|; N \| 14 \| 105 \| | | | |
| --- | --- | --- | --- |
| LV IVS Thickness, cm (median [IQR]) | 1.5 [1.49–1.6] | 1.7 [1.55–2] | 0.004 |
| LV PW Thickness, cm (median [IQR]) | 1.02 [0.89–1.18] | 1.18 [1–1.3] | 0.024 |
| Patients with IVS below 1.5 cm, N (%) | 4 (28.6%) | 9 (8.6%) | 0.047 |
| IVS to PW ratio | 1.53 [1.4–1.77] | 1.45 [1.26–1.78] | 0.578 |
| LV EDD cm (median [IQR]) | 4.36 [3.96–4.87] | 4.40 [4–4.98] | 0.875 |
| LV ESD cm (median [IQR]) | 2.65 [2.49–3.2] | 2.70 [2.36–3.07] | 0.597 |
| LAVI (ml/m^2^) (median [IQR]) | 47 [34.75–60] | 49.5 [37.25–63.75] | 0.707 |
|  |  |  |  |
